# The Active Management of Impending Cephalopelvic Disproportion in Nulliparous Women at Term: A Case Series

**DOI:** 10.1155/2010/708615

**Published:** 2010-07-27

**Authors:** James M. Nicholson, Lisa C. Kellar

**Affiliations:** ^1^Department of Family Medicine and Community Health, University of Pennsylvania Health System, Philadelphia, PA 19104, USA; ^2^Department of Family Medicine, Boonshoft School of Medicine, Wright State University, 101 Wyoming Street, Dayton, OH 45409, USA

## Abstract

*Background*. The Active Management of Risk in Pregnancy at Term (AMOR-IPAT) protocol has been associated in several studies with significant reductions of group cesarean delivery rate. Present within each of these studies were nulliparous women with risk factors for cephalopelvic disproportion. Risk factors for cephalopelvic disproportion in nulliparous women are especially important because they represent the precursors for the most common indication for primary cesarean delivery. *Cases*. Three examples of exposure of urban nulliparous women to the AMOR-IPAT protocol are presented. Each woman's risk factor profile for Cephalopelvic Disproportion (CPD) was used to estimate her Upper Limit of Optimal Time of Vaginal Delivery for CPD (UL-OTDcpd). Labor management and clinical outcomes for each case are presented. A simple table summarizing induction rates and birth outcome rates of exposed versus nonexposed nulliparous women is also presented. *Conclusion*. Because the mode of delivery of the first birth substantially impacts birth options in later pregnancies, the impact of AMOR-IPAT on nulliparous patients is particularly important. Determining the UL-OTDcpd in nulliparous patients, and carefully inducing each patient who has not entered labor by her UL-OTDcpd, may be an effective way of lowering rates of cesarean delivery in nulliparous women.

## 1. AMOR-IPAT in an Urban Setting: A Four-Part Case Series

Over the past two decades, national cesarean section rates have risen dramatically [1]. In 2004, this rate increased to 29.1% [2]. Despite the fact that cesarean section deliveries are associated with increased risk of intra- and postpartum complications for both mothers and babies [3], no strategy to prevent cesarean delivery has been developed. We recently completed two urban retrospective studies that demonstrated strong associations between exposure to an alternative method of care, called the Active Management of Risk in Pregnancy at Term (AMOR-IPAT), and very low cesarean delivery rates [4, 5]. AMOR-IPAT uses risk-driven prostaglandin-assisted preventive induction of labor to promote delivery before prenatal risk can develop into the two major indications for primary cesarean delivery: cephalopelvic disproportion (CPD) and uteroplacental insufficiency (UPI). In these studies of AMOR-IPAT, rates of primary cesarean delivery in both nulliparous and multiparous women, for both CPD and UPI, decreased. The prevention of primary cesarean delivery is especially important because the mode of delivery strongly impacts both the outcomes of the index pregnancy and the management and outcome of future pregnancies [6, 7]. 

In each paper of this four-part series, we present three cases that outline the prenatal risks, clinical management, and birth outcomes of patients exposed to AMOR-IPAT. This paper, the first of the series, focuses on nulliparous women with risk factors for CPD. The second paper will focus on nulliparous women with risk factors for UPI, the third on multiparous women with risk factors for CPD, and the fourth on multiparous women with risk factors for UPI. At the end of each paper, we also present a table summarizing our urban experience with AMOR-IPAT in each parity/risk setting. We hope that these papers will shed some light on the inner workings of AMOR-IPAT and its potential to reduce, in a safe and preventive fashion, primary cesarean delivery rates.

## 2. Introduction to the Prevention of Cephalopelvic Dispropotion in Nulliparous Patients

Primary cesarean delivery is more common in nulliparous than multiparous women, and the mode of delivery of the first birth clearly has a major impact on future pregnancies. Women who undergo cesarean sections with their first birth are more likely to have future pregnancies complicated by placenta previa, spontaneous third-trimester fetal demise, uterine rupture, and/or repeat cesarean delivery [6, 7]. In addition, increasing rates of complications with repeat cesarean delivery have been associated with increasing number of previous cesarean deliveries [6].

The most common indication for primary cesarean delivery in nulliparous women is cephalopelvic disproportion (CPD) [8]. CPD usually refers to the condition where the fetal head is too large to fit through the maternal pelvis. With regards to CPD, AMOR-IPAT preventive theory is based on two main issues. First, while any given woman's pelvis is a set size, the size of her fetus's head continuously grows during the term period of pregnancy; therefore, the risk of CPD continuously increases during the term period. Second, a variety of risk factors predict that either the maternal pelvis is smaller than average (short stature (<62′′) or narrow birth canal noted at initial pelvic exam), the fetus is larger than average (excess weight gain during pregnancy (>30 lb), gestational diabetes, size > dates (≥3 cm), or third trimester ultrasound with a projected EDC that is earlier than previously established EDC), or the fetus will be large relative to the mother's stature (elevated BMI (>30) at conception).

AMOR-IPAT combines these two main issues with a simple scoring system [4, 9]. Most risk factors for CPD have an established odds ratio that quantifies its impact on cesarean delivery risk. Using the AMOR-IPAT paradigm, these odds ratios can be converted into a number of days [4, 9], and the cumulative number of days reflecting each patient's CPD-related risk profile is subtracted from 41 weeks 0 days gestation to estimate a CPD-related upper limit of the optimal time of delivery (UL-OTDcpd). If only one or two CPD risk factors are present, then there is only a modest reduction in the UL-OTDcpd below 41 weeks 0 days of gestation. If there are multiple CPD risk factors present, then the UL-OTDcpd may be reduced to as low as 38 weeks 0 days of gestation. In either case, if spontaneous labor has not started on or before the UL-OTDcpd, then preventive labor induction is recommended. If a patient scheduled for preventive induction has an unfavorable uterine cervix (modified Bishop's score <6), she is offered cervical ripening prior to the use of uterine contractile agents. Especially in the nulliparous patient with an unfavorable cervix, AMOR-IPAT requires a persistent but patient approach to labor induction.

Three cases follow that illustrate the use of AMOR-IPAT in the setting of nulliparous women with risk factors for CPD. These cases are followed by Table 1 that contains summary information concerning rates of labor induction, prostaglandin usage, and cesarean delivery in nulliparous women with risk factors for CPD in the first two urban studies of AMOR-IPAT. In the first urban AMOR-IPAT study, 22 of 30 (73.3%) nulliparous women exposed to AMOR-IPAT had risk factors for CPD compared to 111 of 141 (78.7%) women in the nonexposed group. In the second urban AMOR-IPAT study, 27 of 34 (79.4%) exposed nulliparous women and 81 of 102 (79.4%) nonexposed women had risk factors for CPD. Clearly, in the AMOR-IPAT exposed group, labor induction and the use of prostaglandin for cervical ripening were used more frequently, and cesarean delivery occurred less frequently.

### 2.1. Case  1

A 25-year-old G1 P0 5′  7′′ woman had a LMP and an early second trimester ultrasound that firmly established her EDC. At conception, she weighed around 150 lbs (estimated BMI 24). However, she had gained 35 pounds by 37 weeks estimated gestational age, and measured 3-4 cm size > dates during several visits in her mid third trimester. Her one-hour 50 gm glucola challenge test was 98 and her third trimester hemoglobin was 10.8 gms/dl. Her ULOTDcpd was calculated to be 41 weeks 0 days − 10 days = 39 w 4 days (6 days for excessive weight gain and 4 days for size > dates). However, between 37 and 38 weeks gestation, she gained over 4 lbs and was noted to have a modest elevation of her blood pressure (132/90, 128/86). Due to the combination of impending CPD and impending pre-eclampsia, she was scheduled for preventive induction at 38 weeks and 2 days estimated gestational age.

She presented to the hospital on the evening prior to her delivery, and her fetus was noted to have a vertex presentation. Her cervix was 1 cm dilated, 25% effaced and −3 station. Following placement of an IV and the documentation of a reactive NST, she received a single dose of dinoprostone per vagina (10 mg pleget) at 10 pm. Uterine contractions started just before 5 am. Her contractions became stronger over the next few hours, and the dinoprostone was removed at 8 am. Her cervical exam was unchanged. One hour after the dinoprostone was removed, a pitocin drip was added to maintain and further augment her contractions. Cervical change started to occur about three hours later, that is, around noontime. Several hours later, she was noted to be 5 cm dilated, and epidural analgesia was started. At around 9 pm, she experienced spontaneous rupture of membranes, producing clear amniotic fluid. Cervical exam at that time revealed 6 cm dilatation, 80% effaced and −1 station. She was completely dilated 2-3 hours later. She made steady progress with pushing, and her blood pressure remained within normal limits. Although her second stage lasted just over three hours, she finally delivered a 7 lb 8 ounce infant spontaneously over a small 1st degree perineal tear. Her estimated blood loss was initially 400 cc's, but she had additional bleeding about a half an hour after delivery due to moderate uterine atony. She required external uterine massage, one dose of IM methergine and additional IV pitocin. Her postpartum hemoglobin was 9.6 mg/dl. Her baby's APGAR scores were 8 at 1 minute and 9 at 5 minutes, and it had an unremarkable normal nursery course. Both the mother and her infant were discharged to home on the second postpartum day in good condition.

We believe that, had she been allowed to gestate past 40 week gestation, she would have had a baby weighing eight pounds or more and would have probably required a cesarean delivery for second stage arrest of labor. This belief is largely due to the prolonged second stage that she experienced and the fact that babies in the late third trimester usually grow 4–6 oz/week [10, 11].

### 2.2. Case  2

A 5′  4′′ G1 P0 woman in her early 20s was known to have severe depression but otherwise had an uncomplicated past medical history. An EDC provided by crown-rump length on an 11-week ultrasound was five days later than an EDC provided by a reliable LMP. However, gestational sac measurement on this first ultrasound suggested an EDC that was six days later than the EDC provided by the fetal crown-rump length. Following a review of this information, her final composite EDC was based on the crown-rump length measurement as this balanced the other two estimates. Her BMI at conception was 34. Her one-hour 50-gram glucola challenge was well within normal limits. However, she had gained 36 lbs by 38 weeks gestation, and, during the early 3rd trimester, she repeatedly measured at least 3 cm “size greater than dates”. Her EDC based on a second trimester ultrasound was 9 days earlier than the previously estimated EDC (i.e., the second ultrasound at 24 weeks 5 days gestation suggested the fetus was 26 weeks size), and her EDC based on a third ultrasound was 13 days earlier than the previously estimated EDC (i.e., the third ultrasound at 32 weeks 3 days suggested the fetus was 34 weeks size). We call this phenomenon an “encroaching EDC”, and it is suggestive of excessive fetal weight gain and impending fetal macrosomia. Consistent with this concept, the third trimester ultrasound suggested that her fetus was in the >95th percentile for weight for gestational age (i.e., >2200 gms at 32 weeks 4 days gestation).

At a prenatal visit at 37 weeks, 1 day her UL-OTD was calculated to be 41 weeks − 12 days = 39 w 2 d (2 days for elevated BMI, 4 days for size > dates, and 6 days for excessive weight gain). The lower limit of her optimal time of delivery (LL-OTD) was estimated to be 38 weeks 0 days gestation. The patient requested elective induction as soon as possible because of the relatively large size of her fetus noted during the third ultrasound. She was offered preventive induction of labor at 38 weeks 1 day gestation due to multiple risk factors for CPD and she accepted this offer.

 She presented to the hospital one week later at 38 weeks 1 days gestation. An NST was reactive, and she had normal vital signs. Her fetus had a vertex presentation, confirmed by ultrasound, and her cervix was 1 cm dilated, 50% effaced, and the presenting part was at −4 station. Due to space problems in the labor and delivery department, her induction could not be started until the following morning, when a single dose of 25 mcg dose of misoprostol was given per vagina. Contractions started two hours later, and cervical change was first noted 5 hours after the start of her induction. By suppertime, her cervical exam was 5 cm dilatation, 70% effacement, and −1 station. Artificial rupture of membranes produced clear amniotic fluid. She refused all analgesics. Over the next hour, her fetal heart tracing revealed mild intermittent late decelerations that were successfully treated with left lateral positioning and oxygen. Her contraction frequency and strength began to fade in the late evening, and IV pitocin was started just before midnight. Thereafter, a regular contraction pattern returned. She was completely dilated by 2 am. She pushed for about an hour and finally delivered an 8 pound 0 ounce infant over a small second degree perineal tear. Her EBL was 350 cc's. Her baby's APGAR scores were 8 at 1 minute and 9 at 5 minutes. She and her infant had unremarkable postpartum courses, and both were discharged to home in good condition on postpartum day #2.

We believe that, had her delivery been delayed for another 1-2 weeks, the infant would have grown another 4–8 ounces [10, 11], and the chance of cesarean delivery for CPD would have been considerably higher. In addition, the presence of late decelerations during this labor suggests that, had her delivery been delayed another 1-2 weeks, with associated placental aging, the likelihood of fetal intolerance to labor requiring a cesarean delivery would have also increased.

### 2.3. Case  3

An 18-year-old G2 P 0010 5′  11′′ female had an uncertain last menstrual period, but a 19 week ultrasound was used to determine her EDC. A second ultrasound at around 27 weeks estimated gestational age suggested an EDC to two days earlier than previously estimated. The patient had a preconception weight of 300 lbs (preconception BMI of 42 kg/m^2^). In the late third trimester, she measured 6 cm size greater than dates. Although she “only” gained a total of 25 lbs during her pregnancy, this quantity was considered excessive given her high starting weight. Her 50-gram glucola screen was 110 mg/dl. Her UL-OTD was calculated to be 41 weeks − 12 days = 39 w 2 d (2 days for elevated BMI, 4 days for size > dates, 6 days for excessive weight gain). Due to concerns about the presence of multiple risk factors, and very significant amounts of each risk factor, she was admitted at 38 weeks 3 days gestation for induction of labor for impending CPD.

 On admission (just after suppertime), her cervical exam revealed 0 cm dilatation, 50% effacement, and −3 station. Her fetus had a vertex presentation. She received a 10-hour course of dinoprostone per vagina (pledget) followed by 8 hours of IV pitocin augmentation. Her cervix appeared unchanged at the end of the first day, and the pitocin was stopped. She had supper and a shower, and a second dose of dinoprostone was placed. Ten hours later, the second dose of dinoprostone was removed, and IV pitocin was restarted. Strong contractions developed by around 10 am. By 11 am, a cervical exam revealed 4 cm dilatation, 80% effacement, and −2 station. Artificial rupture of membranes revealed clear amniotic fluid. An epidural catheter was placed for analgesia. Although some mild variable decelerations were noted, the fetal heart rate demonstrated good general variability. The patient continued to make slow progress. After achieving full cervical dilatation, she pushed for about one hour. With the fetal head on the perineum, several deep variable decelerations were noted. A soft vacuum-assisted vaginal delivery occurred, producing a 7 lb 4 oz infant. A first-degree perineal tear was noted and repaired. Her EBL was 700 cc's, and this excessive loss resulted from the laceration of several labial varicosities. Her baby's APGAR scores were 8 at 1 minute and 9 at 5 minutes, and it had an unremarkable normal nursery course. Although the patient's postpartum hemoglobin was 8.1 mg/dl, she remained asymptomatic, and both the patient and her infant were discharged to home in good condition on the second postpartum day.

Again, we believe that, had the infant been born several weeks later, it would have been 8–12 oz heavier [10, 11], and the likelihood of an uncomplicated vaginal delivery would have been substantially reduced.

**Comment**

The use of AMOR-IPAT in nulliparous patients with risk for CPD focuses on ensuring that labor develops before the fetus has become too large to pass through the maternal pelvis. According to AMOR-IPAT theory, the more risk for CPD any given patient has, the earlier labor should occur within the term period in order to maximize the likelihood of an uncomplicated birth.

Especially in nulliparous women, a frequent impediment to the goal of an uncomplicated vaginal delivery is the presence of an unfavorable uterine cervix. We believe that PGE2 products are ideally suited for managing this potential impediment because they generally promote cervical ripening more than uterine contractility and this allows cervical ripening to occur before the onset of active labor. Other methods of cervical ripening (PGE1, foley bulb catheters and laminaria) are also available. Our cases illustrate that the successful induction of a nulliparous woman with an unfavorable cervix often requires the investment of significant time on the part of both the patient and her providers. In approximately half of these inductions, multiple days and multiple doses of PGE2 were needed. However, this investment yields shorter overall hospital length of stay for mother and her baby (due to reduced rates of cesarean delivery and NICU admission) as well as reduction in levels of major adverse birth outcomes. We have found that inductions lasting over 24 hours are not associated with increased infection rates (maternal fever, chorioamnionitis, or suspected neonatal sepsis) as long as amniotomy is not performed until the patient is at least 3 cm dilated and has achieved a modified cervical Bishop's score of at least 6.

These cases illustrate several other important points. First, we have seen a reduction in rates of 3rd and 4th degree perineal injury in patients with risk factors for CPD who are exposed to AMOR-IPAT. This is true for exposed patients who delivered following induction of labor before their UL-OTDcpd and for exposed patients who delivered following the spontaneous onset of labor before their UL-OTDcpd. Of note, the two primary studies that these cases were drawn from showed slightly higher rates of operative vaginal delivery in the exposed groups and so the lower rates of major perineal injury in the exposed groups must have been the product of some other factors. We believe that the other factors were a lower average birth weight and lower average fetal head circumference in infants who deliver before, as compared to after, their mothers' estimated UL-OTDcpd. Second, we have found that our group rates of thick meconium at rupture of membranes have been unusually low. Recent studies have confirmed that the presence of meconium at rupture of membranes is a risk factor for adverse neonatal outcomes [9, 12]. Accordingly, if lower rates of thick meconium passage at rupture of membranes is a marker for improved uteroplacental health, then the lower rates of thick meconium passage seen with the use of AMOR-IAPT represents a secondary benefit from delivery relatively early in the term period of labor. Third, if pregnancy dating has been well established with ultrasound, we do not rely on amniocentesis to confirm fetal lung maturity if preventive induction is performed after 37 weeks 6 days estimated gestational age. In patients preventively induced between 38 week 0 days and 38 week 6 days estimated gestational age, we have not seen increased rates of either NICU admission or problems related to fetal lung immaturity. Fourth, the use of prostaglandins in the setting of preventive induction seems to be associated with a slight increase in the risk of postpartum uterine atony and higher postpartum blood loss. For this reason, we recommend active management of the third stage of labor in all patients (pitocin at delivery of the anterior shoulder, heightened surveillance of uterine tone in the immediate postpartum period, and a lower threshold for the use of methergine and/or hemabate. Finally, the use of AMOR-IPAT has been linked in a small randomized clinical trial with improved birth outcomes, including a lower Adverse Outcome Index (AOI) Score and a higher rate of uncomplicated vaginal delivery [13].

Over the past six years, several physicians in the Department of Family Medicine and community health at the Hospital of the University of Pennsylvania have been using an innovative, alternative method of obstetric care called the Active Management of Risk in Pregnancy at Term (AMOR-IPAT). This method of care advocates preventively inducing pregnant women according to the presence of risk factors that are associated with cesarean delivery and/or morbidity. This management reflects Caughey and Musci's statement [14] that “complications of pregnancy increases not as a discrete risk beyond some arbitrary gestational age, but instead increase continuously as a function of increasing gestational age.” While the benefits and risks of AMOR-IPAT will ultimately need to be determined through a series of randomized controlled trials, our retrospective data strongly suggests that a preventive approach to the improvement of birth outcomes is possible. We hope that these cases clarify some of the methods used in the utilization of AMOR-IPAT in nulliparous patients with risk factors for CPD.

## Figures and Tables

**Table 1:** Summary table of Nulliparous patients* in the two completed Urban Studies [4, 5].

Variables	First urban study: exposed nulliparas* (*n* = 22)	First urban study: nonexposed nulliparas* (*n* = 111)	Second urban study: exposed nulliparas* (*n* = 27)	Second urban study: nonexposed nulliparas* (*n* = 81)
Number/Percent that had labor induction	14/22 (63.6%)	37/111 (33.3%)	15/27 (55.6%)	17/81 (21.0%)

Number/Percent that received PGE2	14/22 (63.6%)	28/111 (25.2%)	14/27 (51.85%)	20/81 (24.7%)

Number/Percent that delivered by Cesarean Section	1/22 (4.6%)	24/111 (21.6%)	5/27 (18.5%)	25/81 (30.9%)


*With risk factors for CPD.
